# Stress and Burnout among Medical Specialists in Romania: A Comparative Study of Clinical and Surgical Physicians

**DOI:** 10.3390/ejihpe14020021

**Published:** 2024-02-01

**Authors:** Ioana Silistraru, Oana Olariu, Anamaria Ciubara, Ștefan Roșca, Anisia-Iuliana Alexa, Florentina Severin, Doina Azoicăi, Radu Dănilă, Sergiu Timofeiov, Ioan-Adrian Ciureanu

**Affiliations:** 1 Faculty of Social Sciences and Humanities, Lucian Blaga University of Sibiu, 550025 Sibiu, Romania; ioana.silistraru@ulbsibiu.ro; 2 Clinical Medical Department, School of Medicine and Pharmacy, Dunărea de Jos University of Galati, 800008 Galati, Romaniaanamaria.ciubara@ugal.ro (A.C.); stefan.rosca@ugal.ro (Ș.R.); 3 2nd Surgical Department, Grigore T. Popa University of Medicine and Pharmacy, 700115 Iasi, Romania; 4 Department of Preventive Medicine and Interdisciplinarity, Grigore T. Popa University of Medicine and Pharmacy, 700115 Iasi, Romania; 5 Department of Surgery I, Grigore T. Popa University of Medicine and Pharmacy, 700115 Iasi, Romania; 6 Medical Informatics and Biostatistics Department, School of Medicine, Grigore T. Popa University of Medicine and Pharmacy, 700115 Iasi, Romania; adrian.ciureanu@umfiasi.ro

**Keywords:** burnout, clinical medical practitioners, surgical specialty, mental health

## Abstract

This study, which focuses on 227 participants (196 females and 31 males) comprising 187 clinical specialists and 40 surgical physicians, examines the prevalence of burnout in medical specialists. We investigate the effects of the emotional exhaustion (EE), Depersonalization (D), and personal accomplishment (PA) dimensions on professional satisfaction and plans to change careers using the modified licensed Maslach Burnout Inventory—Human Services Survey (MBI-HSS (MP)). High emotional exhaustion is reported by 52.63% of men and 71.28% of women in the clinical medicine group (*n* = 188). In the surgical specialties (*n* = 39), scores are significantly higher, with 75% of men and 77.77% of women reporting EE. In our sample group, 71.36% express high levels of emotional exhaustion, with similar patterns across specialization and gender. Clinical medicine respondents report high degrees of depersonalization in 33.13% of females and 21.05% of males, while surgical specialty respondents report high levels in 33.33% of females and 50% of males. Across genders and specializations, 33.03% of all respondents show high levels of depersonalization. Clinical medicine participants report high levels of personal accomplishment (42.60% of females and 42.10% of males), whereas surgical specialties report 44.44% of females and 66.66% of male on the PA dimension. Of the total number of respondents, 44.05% report having a high level of personal accomplishment; differences exist depending on specialty and gender. In addition, questions regarding professional fulfilment and intention to change careers were presented to the participants. A total of 53.40% (16 male and 105 female) of the clinical medicine respondents said they intended to change careers, while 33 participants (9 male, 34 female) doubted if they would remain in the same specialization. Furthermore, 86 individuals (9 male and 77 female) in the surgical specialties said they would never choose to work in healthcare again. Regression analysis suggests that being male, higher in age, and working in surgical specialties with lower job satisfaction and a higher intention to change profession are associated with higher levels of EE. Age and work satisfaction are significant predictors of depersonalization, and higher career satisfaction is associated with increased levels of PA.

## 1. Introduction

Burnout has emerged as a serious concern within the medical profession [1,2,3], affecting healthcare practitioners’ well-being and, as a result, variable quality of patient care. This study examines the complex dynamics of burnout among medical professionals, with a special emphasis on gender gaps, specialty-specific variances, and the potential escalation of burnout amid the COVID-19 crisis [4,5,6], as well as the impact on professional satisfaction and intention to change careers due to the reported burnout.

Burnout rates among physicians and surgeons have risen alarmingly in the global healthcare scene, prompting a careful evaluation of contributing factors and specific patterns within the medical community. Numerous studies have highlighted the pervasiveness of burnout, with consequences ranging from decreased job satisfaction to reduced patient safety [7,8,9]. The Maslach Burnout Inventory—Human Services Survey Medical Personnel (MBI-HSS (MP)) offers a standardized model for evaluating burnout across three dimensions: emotional exhaustion (EE), depersonalization (D), and personal accomplishment (PA) [10,11,12].

Especially since the COVID-19 crisis, researchers have conducted relevant studies on burnout among medical professionals, exploring its diverse traits and varying influence on various specialties. The literature stresses the importance of recognizing burnout as a complex interplay of human, organizational, and environmental influences [13,14,15,16]. Despite significant advancement in understanding the intricacy of burnout, there is still an insufficient number of studies that extensively evaluate burnout patterns based on gender and medical specialty.

Furthermore, the unique challenges resulting from the COVID-19 crisis have complicated the mental health picture for healthcare practitioners [17,18,19,20]. Physicians and surgeons at the forefront of pandemic management have faced novel difficulties, including increased workload, moral dilemmas, and the constant risk of infection. The combination of circumstances raises serious questions regarding the impact of burnout on healthcare staff both during and after the COVID-19 crisis [21,22,23,24]. This study explores the significant impact on career intentions and burnout factors. A significant percentage of respondents in clinical medicine indicate that they plan to change jobs, highlighting the need for interventions aimed at the root causes of dissatisfaction. Many responders in surgical specialties indicate that they have decided to leave the healthcare industry entirely, raising concerns regarding the mechanisms influencing this considerable attrition.

Considering these factors, our study seeks to add to the existing body of knowledge by conducting a comprehensive assessment of burnout levels among medical professionals. We investigate burnout across multiple dimensions, examining gender and specialty-specific variations. Furthermore, we aim to understand how the COVID-19 crisis has affected the mental health of physicians and surgeons, providing insight into the special challenges that healthcare professionals face in Romania [16,25,26]. 

## 2. Materials and Methods

### 2.1. Study Design

In this cross-sectional study, we used an online adaptation of the Maslach Burnout Inventory—Human Services Survey (Medical Personnel) (MBI-HSS (MP)) to assess burnout dimensions such as emotional exhaustion, depersonalization, and personal accomplishment among 227 participants from clinical medicine and surgical specialties. Additionally, we measured professional satisfaction by incorporating an added question to the MBI instrument, which queried participants about their satisfaction with their chosen medical field on a Likert scale from 1 (extremely unsatisfied) to 7 (very satisfied). Regarding intention to change current career, we incorporated additional questions with yes-and-no answer options as follows: “Do you intend to change profession?”; “Would you be choosing the same specialty again?”; and “Would you be choosing medical profession again if given the chance?”. This study, conducted on 26 September 2022, was voluntary and anonymous, according to an authorization letter from Mind Garden by Mrs. Oana Olariu. The licensed survey in English was administered using the Mind Garden platform, which was found appropriate for our sample, minimizing any challenges or sociocultural barriers. To prevent duplicate responses, a one-time-only accessible form link was used to protect data integrity. When participants clicked the survey link, they were informed that their participation was voluntary and they would remain completely anonymous.

### 2.2. Participants and Setting

**Figure 1:**
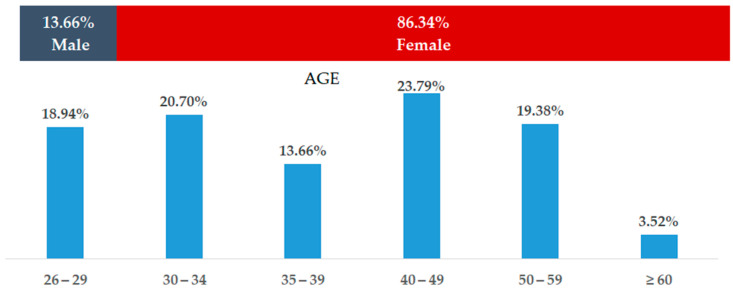
Gender demographics.

Our study is based on a sample group of 227 participants, with a mean age across respondents of 40.15 years, with an SD of 10.22 of age variation (Table 1, Figure 1). The youngest participant was 26 years old, and the maximum age from our sample group was 65. Regarding gender distribution, our sample group consisted of 196 females (86.34% of the total participants) with a mean age of 40.15 years. The minimum age in the female group was 26, while the maximum was 65. As for the male participants, we had 31 (13.65% of the total number of respondents), with a mean age across the male group of 38.35 years, where the minimum age was 26 and the maximum age was 62 years.

In our sample group of *n* = 187 clinical specialties (Table 2), we have the following demographic descriptives: 168 female and 19 male participants; the mean age for female respondents in clinical specialties was 40.42 years, while for males, it was lower at 38.47 years. The overall mean age for both genders combined was 40.22 years. The minimum and maximum ages in both female and male groups were 26 and 65, respectively. 

In our sample group of *n* = 40 surgical physicians, we have the following descriptive data: 28 female and 12 male respondents, with a mean age for female participants of 38.53 years and for male respondents of 38.16 years. The overall mean age for both genders was 38.42 years (Table 3). The minimum and maximum ages in both female and male groups were 27 and 61 for females and 28 and 62 for males, respectively. 

**Figure 2:**
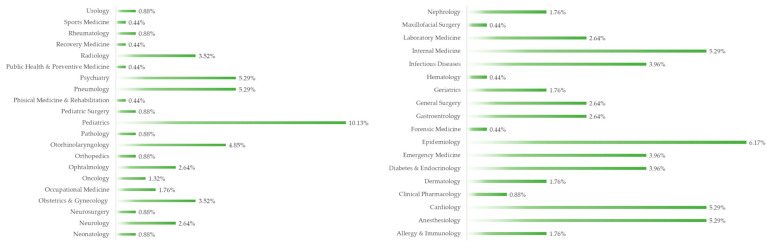
Specialty distribution.

Also, for our sample group, we have the following top five specialty distribution (Figure 2): 22 (10.13%) pediatric; 14 (6.17%) epidemiology; 12 (5.29%) cardiology, psychiatry, pulmonology, internal medicine, and anesthesiology; 10 (4.85%) otorhinolaryngology; and 9 (3.96%) infectious diseases.

### 2.3. Data Collection

To analyze the patterns of burnout within a cohort of 227 individuals, data collection began in November 2021 with the Maslach Burnout Inventory—Human Services Survey Medical Personnel (MBI-HSS MP) licensed instrument. We obtained a diverse participant group for this study, which included 86.34% females and 13.66% males from clinical medicine and surgical specialties. 

### 2.4. Ethical Considerations

Participants received explicit information about this study’s objectives and could opt out during data collection. They were notified about their voluntary involvement by clicking on the MBI-HSS (MP) licensed questionnaire. 

## 3. Results

### 3.1. Burnout Subscales Results 

Our study results suggest that based on a detailed breakdown of burnout subscales among medical professionals in clinical medicine and surgical specialties, discussed by gender, the chi-square (χ^2^) values allocated to each category for comparison indicate the statistical significance of the observed differences (Table 4). 

The general trends in our data suggest that across all clinical specialties (*n* = 187), 71.37% of the respondents experience high emotional exhaustion (EE), whereas surgical specialties (*n* = 40) report a slightly higher percentage (78.57%) of high emotional exhaustion levels. As for the gender differences, among female participants in clinical specialties, 72.02% report high EE, while 52.63% of males report high levels of EE. In surgical specialties, 78.57% of females and 75% of males report high emotional exhaustion. The chi-square test for gender differences on the emotional exhaustion dimension is significant in clinical specialties (*p* = 0.148), suggesting an important gender-related variance. As for the moderate and low levels of EE, these are present in both clinical and surgical specialties in varying percentages. 

Regarding the depersonalization (D) scale results, in our sample group, 33.04% of participants report high depersonalization, whereas the percentage is slightly higher in surgical specialties (35.71%). Among females in clinical specialties, 32.74% report high depersonalization, while 21.05% of males report high depersonalization. In surgical specialties, 35.71% of females and 50.00% of males report high depersonalization. The chi-square test values for gender differences in depersonalization are not significant in clinical specialties (*p* = 0.582) but are borderline significant in surgical specialties (*p* = 0.111). The moderate and low levels of depersonalization vary across genders and specialties.

Our results on the personal accomplishment (PA) burnout dimension suggest that overall, 44.05% of participants report high personal accomplishment (PA), and the values are higher in surgical specialties (66.67%). A total of 42.26% of female respondents in clinical specialties report high PA, while 42.11% of male respondents report the same high PA dimension. In surgical specialties, 46.43% of females and 66.67% of males report high personal accomplishment levels. The chi-square test for gender differences in PA is not significant in clinical specialties (*p* = 0.850); however, the values are borderline significant in surgical specialties (*p* = 0.143). Moderate and low levels of PA vary across genders and specialties.

The descriptive analysis of burnout dimensions and medical specialties suggests that medical specialists experience various levels of burnout across specialties, whereas the burnout subscale data suggest good internal consistency (Table 5).

### 3.2. Emotional Exhaustion (EE)

The mean EE score across all specialties is 34.11, suggesting a relatively high level of emotional exhaustion within our sample group. Clinical specialists have a mean EE score of 33.75, whereas surgical specialists have a higher mean value of 35.75. The 0.888 Cronbach’s alpha value indicates a high internal consistency, as the item measuring emotional exhaustion is reliable and consistent as a construct.

### 3.3. Depersonalization (D)

The mean of the depersonalization score is 10.75 within the sample group, suggesting a moderate level of depersonalization; clinical specialists display a mean D score of 10.44 and surgical respondents score a higher mean value of 12.20. The Cronbach’s alpha value for depersonalization is 0.791, indicating good internal consistency and reliability for measuring the construct.

### 3.4. Personal Accomplishment (PA)

The mean PA score is 31.33, suggesting a relatively high level of personal accomplishment; clinical specialists have a mean PA score of 31.77, while surgery specialists have a slightly lower mean score of 29.30. The Cronbach’s alpha score for PA is 0.824, suggesting good internal consistency, as items measuring the construct are to be considered reliable and consistent.

### 3.5. Burnout and Intention to Change Profession

We compared results within the data set regarding the desire to change professions in our data set, in clinical representatives versus respondents with surgical specialties. In our total sample group who answered “yes” to the question regarding the intention of changing profession (*n* = 121), we had 100 responses from the clinical specialty and 21 from the surgical area, with 16 male (13.22%) respondents and 105 female (86.78%) respondents. The mean age for all groups is 39.28 years with an SD value of ±9.66 within an age range of 26 to 63 years. The mean age for female respondents is 39.40 years, with an SD value of ±9.91 within the age range from 26 to 63 years. The mean age for male participants who responded “yes” to the intention to change profession question is 38.56 years with an SD value of ±8.03 within the age range from 30 to 62 years.

The group considering a different specialty was 43 respondents (33 clinical, 10 surgical), with 9 male respondents (20.93%) and 34 female respondents (79.07%). The mean age for the group is 40.65 years with an SD value of ±9.37 within an age range from 28 years to 62). The mean age for female respondents is 40.6 and an SD value ± 9.53, with the youngest respondent at 28 years and the oldest at 61. The mean age for male respondents within this group is 40.77 years, with an SD value of ±9.32, with the youngest respondent at 30 years and the oldest at 62.

Within the group not choosing the medical field if planning to change profession, we have 86 respondents (73 from clinical specialties and 13 from the surgical field), out of which there are 9 male respondents (10.47%) and 77 female respondents (89.53%). The mean age for all groups is 39.29 years, with an SD value of ±9.83 within the age range from 26 to 63 years. The mean age for female respondents is 39.74 years, with an SD value ±10.19, with the youngest respondent at 26 years and the oldest at 63 years. The mean age for male respondents within the group is 35.44 years, with an SD value of ±4.66, with the youngest respondent at 30 years and the oldest at 45.

Age and gender correlations with burnout dimensions (Table 6) suggest that age has a positive correlation with the emotional exhaustion dimension (0.174) and depersonalization (0.150) and a positive correlation with personal accomplishment (0.115). As for the gender data, there is a positive correlation with EE (0.122), a negative correlation with D subscale (−0.051), and a positive correlation with PA (0.075).

The correlation matrix on burnout dimensions and the intention to change profession and professional satisfaction (Table 7) suggests that emotional exhaustion positively correlates with depersonalization (r = 0.697, *p* < 0.01) and establishes a negative correlation with personal accomplishment (r = −0.476, *p* < 0.01). Our results suggest higher EE is associated with increased depersonalization and a decreased sense of PA. The depersonalization dimension has a significant positive correlation with EE (r = 0.697, *p* < 0.01) and a significant negative correlation with personal accomplishment (r = −0.613, *p* < 0.01), which describes high levels of depersonalization associated with increased emotional exhaustion and decreased personal accomplishment. Also, PA is negatively correlated with EE (r = −0.476, *p* < 0.01) and D (r = −0.613, *p* < 0.01), suggesting that higher personal accomplishment is associated with decreased emotional exhaustion and depersonalization. 

Concerning the intention to change from the medical profession, our data suggest a positive correlation between the emotional exhaustion (r = 0.380, *p* < 0.01) and depersonalization (r = 0.209, *p* < 0.01) subscales and a negative correlation with the personal accomplishment dimension (r = −0.128, *p* < 0.05). While considering a change in profession is associated with higher emotional exhaustion and depersonalization, it is also associated with a lower sense of personal accomplishment. 

The “professional satisfaction” variable is negatively correlated with EE (r = −0.362, *p* < 0.01) and D (r = −0.315, *p* < 0.01), while it presents a positive correlation with PA (r = 0.260, *p* < 0.01). The results suggest that higher professional satisfaction is associated with lower emotional exhaustion and depersonalization and higher personal accomplishment. 

### 3.6. Dependent Variables EE, D, and PA in A Regression Model

Based on the data within the regression model where EE is a dependent variable (Table 8), we suggest that age, gender, specialty, satisfaction, and intention to change profession are significant predictors of emotional exhaustion among medical specialists. As far as age is concerned, the standardized beta coefficient of 0.209 suggests a moderate positive relationship between age and emotional exhaustion, and the beta coefficient for gender of 0.127 indicates a modest positive relationship with EE. The coefficient for medical specialty is 2.960, suggesting that in surgical specialties, physicians have a higher predicted EE score than clinical specialties. Also, the beta coefficient of 0.089 indicates a modest positive relationship. Regarding professional satisfaction, the coefficient is −2.529 and the standardized coefficient beta is −0.250, suggesting a moderate negative relationship between professional satisfaction and EE. As far as the intention to perform a complete professional change, the coefficient for change is 7.264, suggesting that on average, individuals with a higher intention to change profession have a higher predicted EE and a beta coefficient of 0.285, suggesting a moderate positive relationship between the intention to change profession and EE. Our T-values indicate that all predictors have a significant relationship with the EE dimension, as suggested by low *p*-values (sig. values less than 0.05). 

The regression analysis with D as a dependent variable (Table 9) suggests that as the coefficient for variable “age” is 0.135, holding all other variables constant, the predicted depersonalization score will increase yearly by 0.135 units. The standardized coefficient (beta) of 0.185 suggests a moderate positive relationship between age and depersonalization. The “gender” variable is −0.978, suggesting that male respondents have a lower predicted depersonalization score by 0.978 units compared to females, holding other variables constant. However, the coefficient is not statistically significant (*p* = 0.478), and the standardized coefficient (beta) is small (−0.045), indicating a weak relationship between gender and depersonalization. Comparing the coefficients for surgical and clinical specialties, the coefficient is 1.490, suggesting that physicians in surgical specialties have a higher predicted depersonalization score by 1.490 units than those in clinical specialties, holding other variables constant. However, this difference is not statistically significant (*p* = 0.233), and the standardized coefficient (beta) is small (0.077), suggesting a weak relationship. Regarding the professional satisfaction within the sample group, the coefficient is −1.646, suggesting that for other variable constants, the predicted depersonalization score decreases by 1.646 units for each unit increase in satisfaction. The standardized coefficient (beta) of −0.280 indicates a moderate negative relationship between satisfaction and depersonalization. The intention to change professions is marked by a 1.519 coefficient, suggesting that individuals with a higher intention to change professions have a higher predicted depersonalization score by 1.519 units, holding other variables constant. However, this difference is not statistically significant (*p* = 0.140), and the standardized coefficient (beta) is modest (0.102), indicating a weak relationship. The T-test scores suggest that in our data set, age (sig. 0.003) and satisfaction (sig. 0.000) are significant predictors of depersonalization, as indicated by their low *p*-values (sig. values less than 0.05).

The regression model with PA as a dependent variable (Table 10) suggests that for our data set, for the age variable with a coefficient of 0.081, the predicted personal accomplishment score increases by 0.081 units for each additional year of age. The standardized coefficient (beta) of 0.088 suggests a weak positive relationship between age and personal accomplishment. As for the gender variable, the coefficient of 1.445 suggests that, on average, males have a higher predicted personal accomplishment score compared to females, holding other variables constant. However, the coefficient is not statistically significant (*p* = 0.419), and the standardized coefficient (beta) is small (0.053), indicating a weak relationship between gender and personal accomplishment. Surgical and clinical specialties, by comparison, have a coefficient of −1.691, which suggests that physicians in surgical specialties have a lower predicted personal accomplishment score by 1.691 units compared to those in clinical specialties, holding other variables constant. However, this difference is not statistically significant (*p* = 0.295), and the standardized coefficient (beta) is small (−0.069), suggesting a weak relationship. The coefficient for satisfaction is 1.774, suggesting that the predicted personal accomplishment score increases by 1.774 units, holding other variables constant. The standardized coefficient (beta) of 0.241 indicates a moderate positive relationship between satisfaction and personal accomplishment. The professional change variable coefficient is −0.375, implying that, on average, respondents with a higher intention to change professions have a lower predicted personal accomplishment score by 0.375 units, holding other variables constant. However, this difference is not statistically significant (*p* = 0.778), and the standardized coefficient (beta) is very small (−0.020), indicating a weak and negligible relationship. The T-test score values indicate that in our data set, only satisfaction (sig. 0.001) is a significant predictor of personal accomplishment.

## 4. Discussion

The findings of our study reveal several critical aspects of stress, burnout, and career considerations among medical specialists, particularly in clinical and surgical specialties in Romania. The intention to change profession, considerations for a different specialty, and the decision to leave the medical field altogether provide valuable insights into the current state of the medical profession in the years following the COVID-19 crisis.

Our data are consistent with the literature findings [26,27] and indicate that a significant percentage of the respondents manifest intentions to remain in the medical profession (53.30%). However, an in-depth exploration reveals that a smaller yet important group of one-fifth of the respondents (20.93%) contemplates changing their specialty. In this subset of data, our findings suggest that the slightly higher mean age indicates that as they progress in their careers, they may reassess their commitment to their initially chosen specialty. This finding is also aligned with the literature, as we see the highlight of the evolving nature of medical careers as physicians advance in their careers [28]. Our results on the intention to leave the medical field suggest that a considerable percentage of respondents (89.53%), predominantly females, express a desire to leave the medical field. In this subset, males have a lower mean age, suggesting that younger male professionals may be more inclined to explore alternative career paths. Our findings are consistent with the literature, stating that healthcare practitioners’ attrition levels are changing due to burnout and the COVID-19 crisis but are not limited to recent years’ professional burden [29].

Our results also suggest that age and gender disparities relate to burnout in a complex association. The study on the Romanian sample group reveals that older physicians might experience slightly higher emotional exhaustion and depersonalization yet simultaneously perceive slightly higher professional accomplishment. These nuanced findings underscore the multifaceted nature of burnout and its relationship with career longevity. The gender variations in burnout dimensions suggest that female physicians appear to experience slightly higher emotional exhaustion but exhibit lower depersonalization and perceive higher professional accomplishment. These findings align with the existing literature on gender differences in stress-coping mechanisms and professional satisfaction [20,28,30].

Our comparative study also focuses on the association between burnout occurrences and specialty of physicians in our sample group. Our results are aligned with the current literature suggesting that surgical specialties appear to report higher emotional exhaustion [6,18,28]. However, this finding is not statistically significant in our data set, suggesting that burnout is a pervasive concern across clinical and surgical domains in Romania, and further studies are needed to assess the specialty-specific stressors.

As the literature suggests, our regression models indicate that higher career satisfaction is associated with lower emotional exhaustion and depersonalization [28,31,32,33]. Burnout risk mitigation needs in healthcare specialists uncover the importance of organizational support and work environment interventions.

Regarding the predictors of burnout, our regression analysis results show that all variables are involved in the process, including age, gender, specialty, job satisfaction, and intention to change profession, and thorough consideration of predictors might contribute to targeted interventions to mitigate the risk. Our results are also consistent with the existing literature [23,34,35,36,37].

In conclusion, this study not only highlights the prevalence of burnout and career considerations among medical specialists but also delves into the nuanced interplay of demographic factors. The findings underscore the need for holistic approaches to address burnout and promote career satisfaction, considering the unique challenges faced by physicians in various stages of their careers and across different specialties.

## 5. Conclusions

In summary, this research highlights the prevalent burnout problems and career considerations among medical professionals and analyzes the complex relationships between demographic variables. The results highlight the need for comprehensive approaches to reduce burnout and improve career satisfaction while also acknowledging the difficulties experienced by physicians in various specialties and career stages. This study’s nuanced burnout patterns are influenced by gender variations, with surgical specialties revealing greater levels of burnout requiring targeted strategies in high-stress settings. The results highlight significant gender disparities in surgical specialties and call for gender-sensitive strategies to address burnout among medical professionals. The need for qualitative research is emphasized by job satisfaction and retention concerns, particularly among females and those thinking about leaving their careers. Also, the limitations of this study concern the cross-sectional design with potential cause-and-effect constraints, and we emphasize the need of future robust design research on healthcare burnout implications.

## Figures and Tables

**Table 1:** Descriptive demographics for all participants.

	Mean Age	CI		Std. Dev.	Std. Er.	Min	Max	Q25	Q75
		−95%	+95%						
Female 196 (86.34%)	40.15	38.71	41.59	10.22	0.730	26	65	31.50	48.50
Male 31 (13.66%)	38.35	34.68	42.02	9.99	1.79	26	62	30.50	43.50
All	39.91	38.57	41.24	10.19	0.676	26	65	31.00	47.00

**Table 2:** Demographic data on age distribution within clinical specialties.

	Mean Age	CI		Std. Dev.	Std. Er.	Min	Max	Q25	Q75
		−95%	+95%						
Female 168	40.42	38.88	41.97	10.13	0.781	26	65	32.00	48.00
Male 19	38.47	33.45	43.49	10.42	2.39	26	65	30.50	43.00
All	40.22	38.76	41.69	10.14	0.742	26	65	32	47.00

**Table 3:** Demographic data on age distribution within surgical specialties.

	Mean Age	CI		Std. Dev.	Std. Er.	Min	Max	Q25	Q75
		−95%	+95%						
Female 28	38.53	34.33	42.73	10.82	2.04	27	61	28.50	49.50
Male 12	38.16	31.97	44.35	9.74	2.81	28	62	30.50	44.50
All	38.42	35.10	41.74	10.38	1.64	27	62	30	46.50

**Table 4:** Burnout subscale levels by gender and specialty.

		Clinical Specialties (*n* = 187)			Surgical Specialties (*n* = 40)			
Subscale		Female (*n* = 168)	Male (*n* = 19)	Chi	Female (*n* = 28)	Male (*n* = 12)	Chi	Total
EE								
	High	121 (72.02%)	10 (52.63%)	0.148	22 (78.57%)	9 (75.00%)	0.970	162 (71.37%)
	Moderate	27 (16.07%)	4 (21.05%)		4 (14.29%)	2 (16.67%)		37 (16.30%)
	Low	20 (11.90%)	5 (26.32%)		2 (7.14%)	1 (8.33%)		28 (12.33%)
D								
	High	55 (32.74%)	4 (21.05%)	0.582	10 (35.71%)	6 (50.00%)	0.111	75 (33.04%)
	Moderate	37 (22.02%)	5 (26.32%)		2 (7.14%)	3 (25.00%)		47 (20.70%)
	Low	76 (45.24%)	10 (52.63%)		16 (57.14%)	3 (25.00%)		105 (46.26%)
PA								
	High	71 (42.26%)	8 (42.11%)	0.850	13 (46.43%)	8 (66.67%)	0.143	100 (44.05%)
	Moderate	44 (26.19%)	4 (21.05%)		4 (14.29%)	3 (25.00%)		55 (24.23%)
	Low	53 (31.55%)	7 (36.84%)		11 (39.29%)	1 (8.33%)		72 (31.72%)

**Table 5:** Descriptive data of burnout dimensions and medical specialties.

	Mean ± SD	Range	Q25	Q75	Cronbach’s Alpha
EE	34.11 ± 12.76	2.00–54.00	26.00	45.00	0.888
Clinical	33.75 ± 12.92	2.00–54.00	25.50	44.00	
Surgery	35.75 ± 12.00	7.00–54.00	28.00	47.00	
D	10.75 ± 7.43	0.00–30.00	5.00	16.00	0.791
Clinical	10.44 ± 7.19	0.00–30.00	5.00	15.00	
Surgery	12.20 ± 8.38	0.00–30.00	5.50	19.50	
PA	31.33 ± 9.30	6.00–47.00	25.50	38.00	0.824
Clinical	31.77 ± 9.05	6.00–47.00	26.50	38.00	
Surgery	29.30 ± 10.27	9.00–47.00	21.00	38.00	

**Table 6:** Age and gender correlations with burnout dimensions.

		EE	D	PA	Age	Gender
EE	Pearson correlation	1	0.697 **	−0.476 **	0.174 **	0.122
	Sig. (2-tailed)		0.000	0.000	0.009	0.066
	N	227	227	227	227	227
D	Pearson correlation	0.697 **	1	−0.613 **	0.150 *	−0.051
	Sig. (2-tailed)	0.000		0.000	0.024	0.442
	N	227	227	227	227	227
PA	Pearson correlation	−0.476 **	−0.613 **	1	0.115	0.075
	Sig. (2-tailed)	0.000	0.000		0.084	0.259
	N	227	227	227	227	227
Age	Pearson correlation	0.174 **	0.150 *	0.115	1	0.061
	Sig. (2-tailed)	0.009	0.024	0.084		0.361
	N	227	227	227	227	227
Gender	Pearson correlation	0.122	−0.051	0.075	0.061	1
	Sig. (2-tailed)	0.066	0.442	0.259	0.361	
	N	227	227	227	227	227

** Correlation is significant at the 0.01 level (2-tailed). * Correlation is significant at the 0.05 level (2-tailed).

**Table 7:** Correlation matrix of intention to change profession, professional satisfaction, and burnout subscales.

		EE	D	PA	Change Profession	Professional Satisfaction
EE	Pearson correlation	1	0.697 **	−0.476 **	0.380 **	−0.362 **
	Sig. (2-tailed)		0.000	0.000	0.000	0.000
	N	227	227	227	227	227
D	Pearson correlation	0.697 **	1	−0.613 **	0.209 **	−0.315 **
	Sig. (2-tailed)	0.000		0.000	0.002	0.000
	N	227	227	227	227	227
PA	Pearson correlation	−0.476 **	−0.613 **	1	−0.128	0.260 **
	Sig. (2-tailed)	0.000	0.000		0.053	0.000
	N	227	227	227	227	227
Change profession	Pearson correlation	0.380 **	0.209 **	−0.128	1	−0.431 **
	Sig. (2-tailed)	0.000	0.002	0.053		0.000
	N	227	227	227	227	227
Professional satisfaction	Pearson correlation	−0.362 **	−0.315 **	0.260 **	−0.431 **	1
	Sig. (2-tailed)	0.000	0.000	0.000	0.000	
	N	227	227	227	227	227

** Correlation is significant at the 0.01 level (2-tailed).

**Table 8:** Predictors for emotional exhaustion.

Model		Unstandardized Coefficients		Standardized Coefficients	t	Sig.
		B	Std. Error	Beta		
1	(Constant)	16.374	8.162		2.006	0.046
	Age	0.262	0.073	0.209	3.580	0.000
	Gender	4.703	2.209	0.127	2.129	0.034
	Surgical_clinical	2.960	1.996	0.089	1.483	0.140
	Satisfaction	−2.529	0.652	−0.250	−3.877	0.000
	Profession change	7.264	1.644	0.285	4.418	0.000

**Table 9:** Predictors for depersonalization.

Model		Unstandardized Coefficients		Standardized Coefficients	t	Sig.
		B	Std. Error	Beta		
1	(Constant)	10.820	5.090		2.126	0.035
	Age	0.135	0.046	0.185	2.956	0.003
	Gender	−0.978	1.378	−0.045	−0.710	0.478
	Surgical_clinical	1.490	1.245	0.077	1.197	0.233
	Satisfaction	−1.646	0.407	−0.280	−4.047	0.000
	Professional change	1.519	1.025	0.102	1.481	0.140

**Table 10:** Predictors for personal accomplishment.

Model		Unstandardized Coefficients		Standardized Coefficients	t	Sig.
		B	Std. Error	Beta		
1	(Constant)	21.039	6.594		3.191	0.002
	Age	0.081	0.059	0.088	1.362	0.175
	Gender	1.445	1.785	0.053	0.809	0.419
	Surgical_clinical	−1.691	1.613	−0.069	−1.049	0.295
	Satisfaction	1.774	0.527	0.241	3.367	0.001
	Professional change	−0.375	1.328	−0.020	−0.282	0.778

## Data Availability

The data presented in this study are available on request from the corresponding author. The data are not publicly available due to license constraints.
